# Prevalence of suicidal behavior in young university students: A systematic review with meta-analysis*

**DOI:** 10.1590/1518-8345.5320.3495

**Published:** 2021-11-08

**Authors:** Marília de Oliveira Crispim, Cândida Maria Rodrigues dos Santos, Iracema da Silva Frazão, Cecília Maria Farias de Queiroz Frazão, Rossana Carla Rameh de Albuquerque, Jaqueline Galdino Albuquerque Perrelli

**Affiliations:** 1 Universidade Federal de Pernambuco, Hospital das Clínicas, Recife, PE, Brazil.; 2 Universidade Federal de Pernambuco, Departamento de Enfermagem, Recife, PE, Brazil.; 3 Faculdade Pernambucana de Saúde, Recife, PE, Brazil.

**Keywords:** Suicide, Suicidal Ideation, Attempted Suicide, Students, Prevalence, Universities, Suicidio, Ideación Suicida, Intento de Suicidio, Estudiantes, Prevalencia, Universidades, Suicídio, Ideação Suicida, Tentativa de Suicídio, Estudantes, Prevalência, Universidades

## Abstract

**Objective:**

to identify the prevalence of suicidal behavior in young university students.

**Method:**

a systematic review with meta-analysis of cross-sectional studies based on the Joanna Briggs Institute proposal, and carried out in the PubMed, Web of Science, Scopus, PsycINFO and LILACS databases and in the Brazilian Digital Library of Theses and Dissertations, with no language or year restrictions. A total of 2,942 publications were identified. Selection, data extraction and methodological evaluation of the studies were performed by two independent researchers. The meta-analysis was performed considering the random effects model.

**Results:**

eleven articles were included in this review. The prevalence variation for suicidal ideation was from 9.7% to 58.3% and, for attempted suicide, it was from 0.7% to 14.7%. The meta-analysis showed a 27.1% prevalence for suicidal ideation in life, 14.1% for ideation in the last year, and 3.1% for attempted suicide in life.

**Conclusion:**

the high prevalence of suicidal behavior, even with the considerable heterogeneity of the studies, raises the need to implement interventions aimed at preventing suicide and promoting mental health, especially in the academic environment.

## Abstract (es)

**Objetivo:**

identificar la prevalencia de conductas suicidas en jóvenes universitarios.

**Método:**

revisión sistemática con metaanálisis de estudios transversales realizados a partir de la propuesta del *Joanna Briggs Institute*, en las bases de datos *PubMed, Web of Science, Scopus, PsycINFO,* LILACS y Biblioteca Digital Brasileña de Tesis y Disertaciones, sin limitaciones de idioma ni año. Se identificaron 2.942 publicaciones. La selección, la extracción de los datos y la evaluación metodológica de los estudios fueron realizadas por dos investigadores independientes. El metaanálisis se realizó considerando el modelo de efectos aleatorios.

**Resultados:**

se incluyeron once artículos en esta revisión. La variación de la prevalencia de ideación suicida fue del 9,7% al 58,3% y la del intento de suicidio fue del 0,7% al 14,7%. El metaanálisis mostró una prevalencia del 27,1% para la ideación suicida a lo largo de la vida, del 14,1% para la ideación en el último año y del 3,1% para el intento de suicidio a lo largo de la vida.

**Conclusión:**

la alta prevalencia de conducta suicida, a pesar de la considerable heterogeneidad de los estudios, plantea la necesidad de implementar intervenciones dirigidas a prevenir el suicidio y promover la salud mental, especialmente en el ámbito académico.

## Abstract (pt)

**Objetivo:**

identificar a prevalência do comportamento suicida em jovens universitários.

**Método:**

revisão sistemática com metanálise de estudos transversais realizada a partir da proposta do *Joanna Briggs Institute*, nas bases de dados *PubMed, Web of Science, Scopus, PsycINFO,* LILACS e Biblioteca Digital Brasileira de Teses e Dissertações, sem restrição de idioma e ano. Foram identificadas 2.942 publicações. Seleção, extração dos dados e avaliação metodológica dos estudos foram realizadas por dois pesquisadores independentes. A metanálise foi realizada considerando o modelo de efeitos randômicos.

**Resultados:**

onze artigos foram incluídos nesta revisão. A variação da prevalência da ideação suicida foi de 9,7% a 58,3% e da tentativa de suicídio foi de 0,7% a 14,7%. A metanálise demonstrou prevalência de 27,1% para ideação suicida na vida, 14,1% para ideação no último ano e 3,1% para tentativa de suicídio na vida.

**Conclusão:**

a alta prevalência do comportamento suicida, mesmo diante da considerável heterogeneidade dos estudos, desperta a necessidade de implementação de intervenções voltadas para prevenção do suicídio e promoção da saúde mental, sobretudo no espaço acadêmico.

## Introduction

The University is a space for political, social and professional training that enables the construction of fundamental knowledge about a given area and favors the development of skills inherent to the professional’s performance. The academic study phase requires making important decisions, in addition to being a time for new experiences, discoveries and friendships cycles that will require the student to be able to deal with new situations. However, unsatisfactory adaptation to this context can lead to mental distress in the life of this young person^(1-2)^.

In addition to these issues, it is highlighted that the academic setting can generate a competitive environment among the students. The requirement to excel, the excessive hour load of academic subjects and work, and unharmonious relationships with the professors can generate tension and overload, as well as trigger mental illness in the student, in addition to contributing to an increased suicide risk^(2)^.

Suicidal behavior is a complex phenomenon that includes ideation, attempted and consummated suicide, and is related to biological, psychological, social and environmental factors^(3)^. Attempted suicide is the manifestation of a process that develops gradually^(4)^. It is necessary to know the circumstances in which suicidal behavior arises to prevent the factors that cause it^(5)^.

Data from the World Health Organization (WHO) evidenced approximately 800,000 deaths due to suicide worldwide in 2016, which represents an annual rate of 10.6 suicides *per* 100,000 inhabitants. In addition, among young people, suicide is the second leading cause of death in the 15-29 year-old age group worldwide, and one of the 10 leading causes of death in North America^(6-8)^. Thus, suicide is a social phenomenon^(9)^, a serious public health problem that needs to be addressed by civil society and by the public administration. It is an avoidable event through the early identification of suicidal behavior and efficient intervention strategies^(8)^. Therefore, it becomes necessary to know the prevalence of this behavior and the factors that are associated with its occurrence.

Identification of the suicide and suicidal behavior rates favors the implementation of strategies to reduce this event^(10)^, which, combined with the recognition of the determining or risk factors for this phenomenon, enables action from a prevention perspective^(11)^, in addition to providing subsidies to devise evidence-based strategies^(12)^.

Several risk factors predispose the individual to a greater chance of developing suicidal behavior^(8-9)^, the following among them: individual (childhood adversities, severe mental disorders, depression, personality disorders, drug abuse, physical health problems) and environmental (violence, socioeconomic inequality, lifestyle, lack of social support, media effects and access to lethal means), which combine and generate greater vulnerability to suicidal behavior^(8-9)^. Therefore, this phenomenon must be understood from a multi-factorial perspective, since considering it only in its biological context makes it impossible for intervention measures to be effective^(12)^. Given the above, the objective of this study is to identify the prevalence of suicidal behavior in young university students.

## Method

### Type of study

This is a systematic review, registered in PROSPERO (International Prospective Register of Ongoing Systematic Reviews) as CRD42020153709, and developed according to the recommendations of the Joanna Briggs Institute Reviewers’ Manual (JBI)^(13-14)^ to report prevalence and incidence systematic reviews and of the Preferred Reporting Items for Systematic Reviews and Meta-Analyses (PRISMA)^(15)^.

The review question was created from the CoCoPop (Condition, Context and Population) mnemonic, according to the JBI^(13-14)^. The condition chosen was suicidal behavior (suicidal ideation and/or attempted suicide and/or suicide), the context and population defined were young university students.

In this review, suicidal behavior was assessed based on suicidal ideation, defined as thoughts and ideas of ending one’s life; with attempted suicide understood as attitudes that cause injuries with the objective of causing self-harm, with the intention of killing oneself, and suicide as the act of ending one’s life^(16-17)^. Thus, the following question was elaborated: What is the prevalence of suicidal behavior (suicidal ideation and/or attempted suicide and/or suicide) in young university students?

Initially, an initial search was carried out in two databases, PubMed/MEDLINE and PsycINFO, as recommended by the JBI^(14)^, followed by the analysis of the words contained in the text, title and abstract in search of the main terms used in the literature. Subsequently, a search strategy was developed for each database. Collection in the databases took place from November to December 2019, and was updated in November 2020.

### Research scenario and data collection period

The study setting consisted of the PubMed/MEDLINE (National Center for Biotechnology Information/Medical Literature Analysis and Retrieval System Online), Web of Science, Scopus, PsycINFO (Psychology Information) and LILACS (Latin-American and Caribbean Center on Health Sciences Information) databases.

To search the CoCoPop mnemonic, we considered the following descriptors/keywords: Condition: “suicidal ideation”; “attempted suicide”; “suicide”; “suicidal behavior”; “suicidality”. Context and Population: “university students”; “undergraduate”; “undergraduate education”; “colleges”; “college students”; “academics” and “students”.

**Figure 1:**
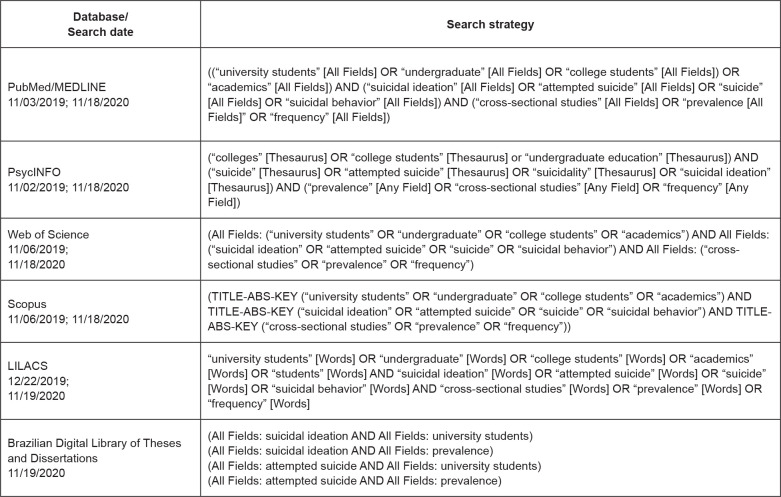
Search strategy used in the respective databases

The search strategy was structured with controlled terms and keywords, according to each database, and is described in Figure 1.

### Publication selection criteria

The publication selection criteria were designed to answer the aforementioned review question. Thus, the following inclusion criteria were adopted: original articles characterized as cross-sectional studies, carried out with undergraduate students aged 18 years old or over, with no restrictions on gender and geographic location; and studies that evaluated the occurrence of suicidal ideation and/or attempted suicide and/or suicide, using a probabilistic sampling technique.

As for the exclusion criteria, the following were adopted: studies that did not detail the age group or that included an age group under and over 18 in the same sample, but did not detail the results by age group that would support the extraction of suicidal ideation and/or attempted suicide for those over 18 years of age; research studies with graduate students; and surveys that did not use a validated instrument to assess suicidal behavior or that presented an incomprehensible methodology.

To expand the number of studies, no date or language restrictions were applied; in addition to that, the reference lists of the eligible studies and in the Brazilian Digital Library of Theses and Dissertations were consulted, whose search strategy is described in Figure 1.

After searching the databases, the publications were exported with the aid of the Zotero reference manager and duplicates were removed.

### Instruments used and research variables

The JBI standardized instrument was used to extract data from prevalence and incidence studies, which contains information about the following: condition measured; measurement method; and characteristics of the participants and of the study^(13-14)^.

In addition, to assess the methodological quality, the JBI checklist for prevalence studies^(13-14)^ was used, composed of nine items whose answers can be: yes, unclear, no and not applicable. It was decided not to exclude articles during the methodological quality assessment stage. The review team discussed each assessment item for each study design included in relation to what was considered acceptable for the review. In order for the process to be transparent, the decision not to establish a cutoff point for inclusion was made before starting the critical assessment and was agreed upon among all the reviewers. This decision is consistent with the JBI guidance in noting that cutoff scores are generally advised against. The JBI recommends presenting the critical evaluation results for all the questions using a table rather than summarizing them with a score, which was presented in the manuscript in the Results section to ensure quality and transparency of the writing.

In this study, in order to assess item 3 of the aforementioned checklist that deals with the adequate minimum sample size for a prevalence study, the JBI recommends the use of sample calculation by means of the following formula:, where Z^2^_α_: 1.96; P (prevalence of the phenomenon of interest): 20.0% or 0.20; and 5.0% (0.05) sample error (d). Thus, studies with a minimum sample of 246 participants were considered adequate.

As for item 5 (Was the data analysis conducted with sufficient coverage of the identified sample?), a minimum sample size of 246 was considered; as well as probabilistic or census selection of the participants in order to minimize selection bias; and a 70.0%^(18-19)^ minimum response rate which, in turn, was also a parameter for item 9.

### Data treatment and analysis

The studies were critically evaluated by two independent researchers. In case of disagreement, a third reviewer was consulted. Initially, titles and abstracts were read and then the text was read in full. Subsequently, the eligible studies were evaluated for their methodological quality, using the JBI Critical Appraisal Checklist for Studies Reporting Prevalence Data instrument^(13-14)^. The instrument evaluates the following criteria^(13-14)^: appropriate recruitment of the participants; adequate sample size; detailed description of the subjects and of the study setting; data analysis with sufficient sample coverage; use of valid methods to identify the condition; measurement of the phenomenon in question in a standard way for all the participants; satisfactory response rate; and adequacy of the statistical analysis.

The evaluation, extraction, synthesis and meta-analysis stages were carried out with the aid of JBI’s System for the Unified Management, Assessment and Review of Information (SUMARI) software. After evaluating the methodological quality, the data were extracted with the aid of the JBI instrument^(13-14)^. In addition, information was collected about the following: data collection locus, gender and age group of the participants, instrument used to assess suicidal ideation, attempted and consummated suicide and prevalence of suicidal behavior, in addition to the statistical analysis technique. It is noted that the first two stages were performed by two independent researchers, previously trained to minimize evaluation and extraction errors.

To perform the meta-analysis, data on the prevalence of suicidal ideation and attempted suicide were organized as follows: ideation in life, during the year and over the last week; attempted suicide in life, during the year and over the last week. Subsequently, the meta-analysis was carried out considering the random effects model.

### Ethical aspects

As this is not a research study involving human beings, it was not necessary to submit it to any Committee of Ethics in Research with human beings.

## Results

**Figure 2:**
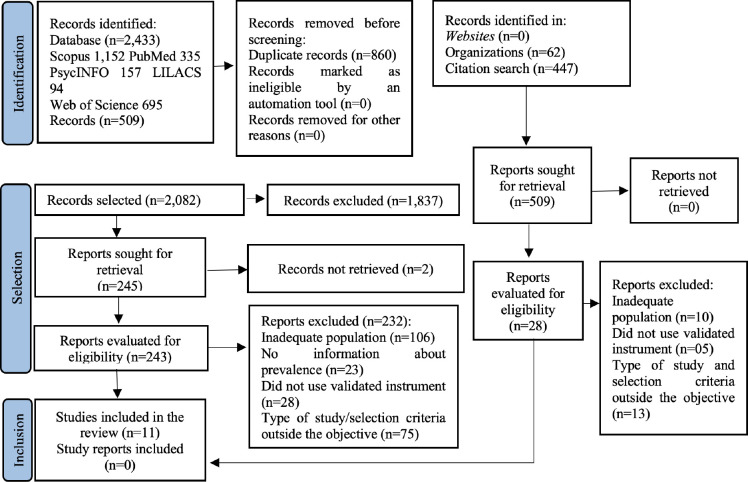
PRISMA Flowchart^(15)^ corresponding to the result of the search, selection and inclusion of studies

A total of 2,942 studies were identified in the databases, records and other methods, of which 860 were excluded for being duplicates and 1,837 for not meeting the eligibility criteria, after reading the titles and/or abstracts. Thus, 245 articles were selected for full-text reading, of which two were not available for access, resulting in 243 articles for full reading. After analyzing the eligibility criteria, 232 studies were excluded, resulting in 11 articles that comprised this review^(20-30)^. Figure 2 presents the flowchart corresponding to the results of the search, selection and inclusion of studies, as well as the reasons for the excluded studies.

The studies included met most of the JBI checklist’s criteria for prevalence studies. However, two research studies did not meet the minimum sample size criterion (n<246); one study did not clearly present the research participants and locus; three presented data analysis with inadequate sample sizes (n<246); and three showed a response rate below 70.0%. It is noteworthy, however, that all the participants were selected in a random/probabilistic or censused manner. Further details are described in Table 1.

**Table 1:** Assessment of the methodological quality of the studies included in this systematic review (n=11). Recife, PE, Brazil, 2021

Author, Year	1*	2^†^	3^‡^	4^§^	5^||^	6^¶^	7**	8^††^	9^‡ ‡^	%
Abdu, 2020^(20)^	Y^§§^	Y^§§^	Y^§§^	Y^§§^	Y^§§^	Y^§§^	Y^§§^	Y^§§^	Y^§§^	100.0
Alexandrino-Silva, 2009^(21)^	Y^§§^	Y^§§^	Y^§§^	Y^§§^	N^||||^	Y^§§^	Y^§§^	Y^§§^	N^||||^	77.8
Benjet, 2019^(22)^	Y^§§^	Y^§§^	Y^§§^	Y^§§^	Y^§§^	Y^§§^	Y^§§^	Y^§§^	Y^§§^	100.0
Galicia, 2019^(23)^	Y^§§^	Y^§§^	N^||||^	UC^¶¶^	Y^§§^	Y^§§^	Y^§§^	Y^§§^	Y^§§^	77.8
Lockman, 2016^(24)^	Y^§§^	Y^§§^	Y^§§^	Y^§§^	N^||||^	Y^§§^	Y^§§^	Y^§§^	N^||||^	77.8
Marraccini, 2019^(25)^	Y^§§^	Y^§§^	Y^§§^	Y^§§^	N^||||^	Y^§§^	Y^§§^	Y^§§^	N^||||^	77.7
Menezes, 2012^(26)^	Y^§§^	Y^§§^	N^||||^	Y^§§^	Y^§§^	Y^§§^	Y^§§^	Y^§§^	Y^§§^	88.8
Miranda-Mendizabal, 2019^(27)^	Y^§§^	Y^§§^	Y^§§^	Y^§§^	Y^§§^	Y^§§^	Y^§§^	Y^§§^	Y^§§^	100.0
Pereira, 2015^(28)^	Y^§§^	Y^§§^	Y^§§^	Y^§§^	Y^§§^	Y^§§^	Y^§§^	Y^§§^	Y^§§^	100.0
Quarshie, 2019^(29)^	Y^§§^	Y^§§^	Y^§§^	Y^§§^	Y^§§^	Y^§§^	Y^§§^	Y^§§^	Y^§§^	100.0
Veloso, 2019^(30)^	Y^§§^	Y^§§^	N^||||^	Y^§§^	Y^§§^	Y^§§^	Y^§§^	Y^§§^	Y^§§^	88.8
%	100.0	100.0	76.9	92.3	69.2	100.0	100.0	100.0	69.2	

* 1 = Was the sample frame appropriate to address the target population?;

† 2 = Were study participants sampled in an appropriate way?;

‡ 3 = Was the sample size adequate?;

§ 4 = Were the study subjects and the setting described in detail?;

|| 5 = Was the data analysis conducted with sufficient coverage of the identified sample?;

¶ 6 = Were valid methods used for the identification of the condition?;

** 7 = Was the condition measured in a standard, reliable way for all participants?;

†† 8 = Was there appropriate statistical analysis?;

‡‡ 9 = Was the response rate adequate, and if not, was the low response rate managed appropriately?;

§§ Y = Yes;

|||| N = No;

¶¶ UC = Unclear

According to data in Table 2, most of the research studies (n=8; 72.7%) presented a high response rate, with the exception of three surveys whose rates were from 55.0% to 61.0%^(21)^, 8.0%^(24)^ and 11.0%^(25)^, respectively. Furthermore, two surveys did not report the percentage of participants who answered the research instruments^(27-28)^. Therefore, calculation for these two studies was performed, based on the expected and achieved sample size disclosed by the authors.

The studies were developed from 2009 to 2020 in North America^(22,24-25)^; South America^(21,30)^; Europe^(27-28)^; Asia^(22,25)^ and Africa^(20,29)^. The sample varied from 142 to 4,189, with a total of 9,511 students. The mean age was approximately 21 years old. The sampling methods used to select the participants were as follows: probabilistic sampling (random/stratified)^(20,23-26,28-30)^ or census^(21-22,27)^. The most used instruments to assess suicidal behavior were the Self-Injurious Thoughts and Behaviors Interview (SITBI)^(22,25,27)^ *and the* Suicidal Behavior Questionnaire Revised (SBQ-R)^(20,29)^. Regarding the prevalence of suicidal behavior, suicidal ideation varied from 9.7% to 58.3%^(20-30)^, and attempted suicide ranged from 0.7% to 14.7%^(20,22-25,27,29)^.

**Table 2:** Characteristics of the studies included and prevalence of suicidal behavior in young university students (n=11). Recife, PE, Brazil, 2021

Author/Year	AG*	n^†^	RP^‡^	I^§^	P^||^	SI^¶^	AS**
Abdu, Hajure, Desalegn, 2020^(20)^	>18	523	100.0	SBQ-R^††^	IL^‡‡^	58.3	4.4
Alexandrino-Silva, Pereira, Bustamante, Ferraz, Baldassin, Andrade, et al., 2009^(21)^	>18	563	55.0-61.0	BSI^§§^	LW^||||^	13.0	NI^¶¶^
Benjet, Gutiérrez-Garcia, Abrego-Ramírez, Borges, Covarrubias-Díaz, Durán, et al., 2019^(22)^	>18	4,189	79.3	SITBI***/ C-SSRS^†††^	IL^‡‡^	23.0	3.5
					LY^‡‡‡^	9.7	0.7
Galicia, Bautista, 2018^(23)^	19-27	225	100.0	DSHI^§§§^	IL^‡‡^	NI^¶¶^	14.7
Lockman, Servaty-Seib, 2016^(24)^	18-25	165	8.0	SIS^||||||^	IL^‡‡^	29.0	4.2
Marraccini, Brick, Weyandt, Francis, Clarkin, Fang, 2019^(25)^	>18	722	11.0	SITBI***	IL^‡‡^	26.0	1.7
Menezes, Subba, Sathian, Kharoshah, Senthilkumaran, Pant, et al., 2012^(26)^	18-27	206	100.0	GHQ-28^¶¶^	IL^‡‡^	18.4	NI^¶¶^
					LY^‡‡‡^	10.7	NI^¶^
Miranda-Mendizabal, Castellví, Alayo, Vilagut, Blasco, Torrent, 2019^(27)^	18-24	2,105	87.9	SITBI***/ C-SSRS^†††^	LY^‡‡‡^	19.7	1.4
Pereira, Cardoso, 2015^(28)^	18-58	366	100.0	SIQ****	LY^‡‡‡^	10.7	NI^¶¶^
Quarshie, Cheataa Plange, Annor, Asare Doku, Lartey, 2019^(29)^	18-35	305	95.0	SBQ-R^††^	IL^‡‡^	15.4	2.3
					LY^‡‡‡^	21.3	NI^¶¶^
Veloso, Lima, Sales, Monteiro, Gonçalves, Silva Júnior, 2019^(30)^	>18	849	100.0	BSI^§§^	LW^||||^	22.0	NI^¶¶^

* AG = Age Group;

† n = Sample size;

‡ RP = Study Response Percentage;

§ I = Measuring Instrument;

|| P = Period;

¶ SI = Suicidal Ideation percentage;

** AS = Attempted Suicide percentage;

†† SBQ-R = Suicide Behaviors Questionnaire Revised;

‡‡ IL = In Life;

§§ BSI = Beck Scale for Suicidal Ideation;

|||| LW = Last Week;

¶¶ NI = Not Informed;

*** SITBI = Self-Injurious Thoughts and Behaviors Interview;

††† C-SSRS = Columbia-Suicide Severity Rating Scale;

‡‡‡ LY = Last Year;

§§§ DSHI = Deliberate Self-Harm Inventory;

|||||| SIS = Suicidal Ideation Scale;

¶¶¶ GHQ-28 = General Health Questionnaire;

**** SIQ = The Suicidal Ideation Questionnaire

Five research studies stratified the prevalence of suicidal ideation by gender^(20,22,25,27,29)^. In relation to ideation in life, prevalence was higher in women, ranging from 16.2% to 28.2%^(20,22,25,27,29)^, when compared to men, whose variation was from 7.1% to 20.5%^(22,25,27,29)^. Only one study^(20)^ presented higher prevalence of suicidal ideation in the male gender (31.0%). Ideation in the last year varied from 10.5% to 21.7% among the women^(22,27,29)^; and from 7.9% to 17.9%^(22,27,29)^ among the men.

As for the undergraduate course, four studies^(21,26,29,30)^ evaluated suicidal behavior in health sciences students, of which two estimated the prevalence in undergraduate Nursing^(21,29)^, two in medical students^(21,26)^ and one in Pharmacy students^(21)^. The suicidal ideation rate in the Nursing course varied from 12.3% (in the last week)^(21)^ to 21.3% (in the last year)^(29)^; for the Medicine course, the variation was from 13.4% (in the last week)^(21)^ to 18.4% (in life)^(26)^; and the ideation rate found in the Pharmacy course (in the last week) was 12.3%^(21)^.

Considering the variability of instruments used, the different sample sizes and the different countries where the studies were developed, it is expected that there will be wide heterogeneity across the studies. However, the main objective of this review was to provide a general overview of the prevalence of suicidal behavior (ideation and attempted suicide); thus, even in the presence of high heterogeneity, it was decided to perform and present the meta-analysis.

**Figure 3:**
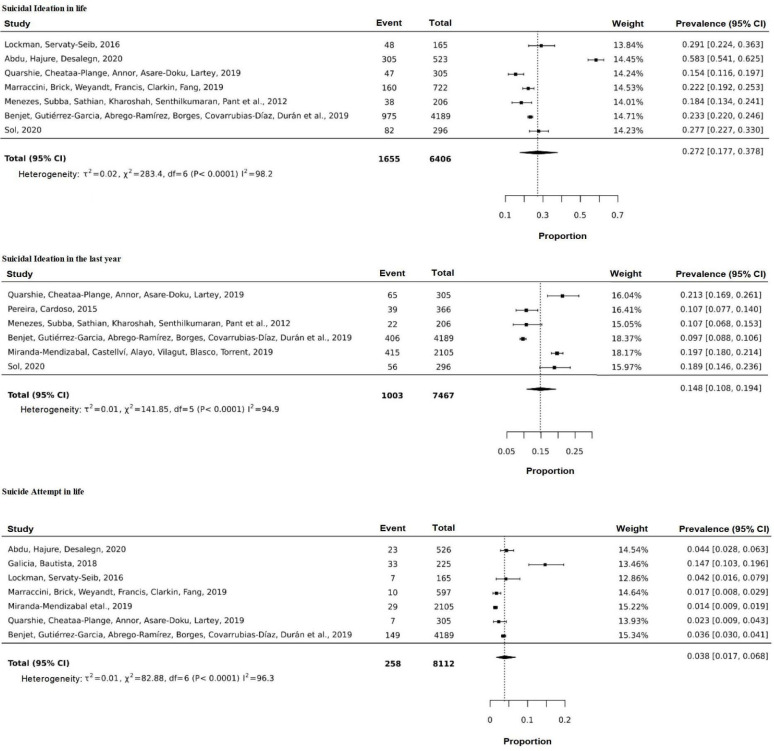
Meta-analysis of the prevalence of suicidal ideation and attempted suicide among young university students

Regarding the results of the meta-analysis (Figure 3), the prevalence values of suicidal ideation in life and in the last year were 27.1% (CI: 16.0 - 39.7; I^2^: 98.5; p<0.0001) and 14.1% (CI: 9.6 - 19.3; I^2^: 95.9; p<0.0001), respectively. As for the prevalence of attempted suicide in life, it was 3.8% (CI: 1.7 - 6.8; I^2^: 96.3; p<0,0001) among young university students.

It was not possible to perform a meta-analysis of suicidal ideation in the week immediately before data collection and of attempted suicide in the last year, since only two research studies reported the percentages of these phenomena in the respective periods cited.

It is noted that the combined prevalence values presented must be evaluated with caution, as high heterogeneity was observed across the studies. However, the data show a worrying panorama of the problem of suicide among young university students, albeit in a descriptive way.

## Discussion

Balanced geographic distribution of the studies included was observed, with predominance of publications in the last ten years. The condensation of studies in this period can be related to the economic crisis and austerity policy, which began in 2008 and caused periods of economic recession, with an increase in the suicide rates^(31)^, which may have prompted the increase in the number of research studies. Economic crises have effects on mental health and can increase the risk factors and weaken the protective factors^(32-33)^.

The prevalence of suicidal behavior varied widely. The study carried out in Ethiopia^(20)^ presented the highest rate of suicidal ideation, with a quite expressive percentage, and the one conducted in Mexico^(22)^ showed a lower percentage. However, when the recall period included the presence of suicidal behavior in life, the rate in Mexico increased significantly.

The ideation rate presented in the study conducted in Ethiopia was higher than the one found in other surveys that evaluated suicidal ideation in young individuals^(34-35)^. The prevalence found was close to studies that evaluated suicidal behavior in patients with mental disorders^(36-37)^, whose ideation rate tends to be higher due to mental illness. There are few research studies on suicidal behavior in Sub-Saharan Africa, which can be related to limited resources, as well as to the stigma associated with suicide in some countries of this region^(38-39)^. In these countries, among them Ethiopia, there is difficulty accessing mental health services, with shortage of professionals and mental health care institutions centralized in capital cities^(38,40)^.

The scarcity of data on suicidal behavior leads to underreporting, increased stigma and difficulty accessing the health system^(38)^. Middle- and low-income countries account for approximately 75.0% of the number of suicides in the world^(7)^; therefore, it is important to recognize the role of the sociocultural and economic factors in suicide, which can be expressed in different ways according to gender, reflecting social roles^(41)^.

Although Mexico presented the lowest rate of suicidal ideation in the last year, when compared to the other surveys, this prevalence increased when referring to suicidal thoughts at some point in life. A study carried out in 2016 with a sample of 56,877 Mexicans evidenced that suicidal behavior is a growing phenomenon seen throughout the country, especially among women, young individuals, single and with lower schooling levels^(42)^. This growing trend can be related to the economic crisis experienced in the last decade and to the increase in exposure to violence^(43-44)^.

The prevalence of suicide ideation and attempted suicide was expressive among women, corroborating results from other studies^(45-46)^. Gender relationships are found throughout the suicidal behavior period, from ideation to attempted suicide. Therefore, the expression of psychological distress is usually different between men and women. Thus, this difference can significantly affect the prevalence of suicidal behavior, which can be related to the exercise of a role that is socially and culturally required from women^(47)^.

The highest prevalence of attempted suicide was shown in the study carried out in the Philippines^(23)^. This result is consistent with a research study that evidenced a 16.4% prevalence of suicide attempt in life and of 4.7% in the last 30 days among high school students^(48)^. Although the Philippines presented relatively low suicide rates when compared to other Southeast Asian countries, most of the countries in this region do not have a comprehensive registration system, which can indicate underreporting^(49)^. In addition to that, the difficulties accessing mental health services and the economic conditions limit the reach of mental health care and the search for help^(50)^.

The comparison between the prevalence values of the studies became difficult, given the methodological differences, variation in the measuring instruments and analyses performed. In the assessment of suicidal behavior, the instruments that were most used in the studies analyzed were SITBI^(51)^ and SBQ-R^(52)^.

SITBI is a structured interview that assesses self-injurious behaviors, which include suicide ideation, plans and attempts, in addition to non-suicidal self-mutilation, which can be used in research and clinical settings. The instrument presented high inter-evaluator reliability and test reliability, in addition to concurrent validity demonstrated by a strong correspondence between SITBI and other suicidal ideation measures, in a sample of adolescents and young adults^(51)^.

The Suicidal Behaviors Questionnaire (SBQ) assesses the extent of suicidal behavior, as well as the risk of suicide or self-harm. Its revised version (SBQ-R) consists of four items that assess suicidal behavior in the last year and in life^(52)^, providing support as a suicide risk measure for use in clinical and non-clinical contexts, with acceptable internal consistency, excellent test-retest reliability, high sensitivity (93.0%) and specificity (95.0%)^(52)^.

The use of valid and trustworthy instruments to measure suicidal behavior is capable of providing reliable results regarding this phenomenon, which, in turn, will support the implementation of interventions for the prevention of suicide. Knowledge of the applicability and validity of these instruments in the Brazilian context can be relevant.

Most of the studies in this review included students from the health sciences, most notably Medicine and Nursing. However, there is evidence of high prevalence of suicidal ideation in students from different knowledge areas, with a percentage of 9.9% in the last 30 days. The associated factors were sexual orientation, suicide attempts in the family and presence of depressive symptoms^(53)^. A study conducted with students from two universities in South Africa found a 3.9% prevalence of attempted suicide, with an increased risk associated with students who identified themselves as “black-skinned” and “female”^(54)^.

Specifically in the health context, a systematic review evidenced an association between depression, depressive symptoms, previous diagnosis of psychiatric disorder, lower socioeconomic level/financial difficulties, history of drug use and feelings of neglect by parents, as factors associated with suicidal ideation in medical students^(55)^.

Depression, anxiety, and suicidal ideation have been reported by health sciences university students after entering their academic programs. This can be related to the rigor and demand of the courses, in addition to the high stress and wear out levels^(56)^. In addition to that, most university students do not seek help to deal with their emotional problems, such as suicidal thoughts, under the allegation of shame and preference to deal with the problem by themselves, which reinforces the need for interventions in academic environments that would help overcome the stigma attached to seeking help^(57)^.

Since these students will be future professionals who will provide health care to other people, their own mental health needs are of particular importance and must be addressed^(56)^. It is known that health professionals are at an increased risk of anxiety, depression and/or suicidal ideation when compared to the general population^(58)^. Thus, early screening for mental distress in the academic setting can reflect positively on their professional life.

It is noted that the variation in the prevalence of suicidal behavior can be related to different instruments used in the assessment, at different periods, in addition to the socioeconomic, continental and cultural aspects. Thus, it is recommended to evaluate the results of the meta-analysis of suicidal ideation and attempted suicide with caution, due to the high heterogeneity across the studies. However, it is noteworthy that the findings are relevant, especially from a descriptive point of view, to signal the seriousness of the problem of suicide and induce strategies to prevent it and promote mental health among young university students.

The publication bias stands out as a limitation of this review, since not all the scientific databases were investigated, as well as the fact that only studies available for free were included. However, some strategies were used to try to reduce the bias regarding the design of the studies that comprised the review, such as the inclusion of research studies that used validated instruments to minimize overestimations or misclassification errors of single-item self-report questions; as well as the inclusion of those that used probabilistic samples to obtain more valid estimates.

## Conclusion

The results of the meta-analysis showed high heterogeneity in the studies included in this review. However, the descriptive findings pointed to a high prevalence of suicidal ideation and attempted suicide in young university students, more pronounced in women. The results show the need to implement health promotion public policies that consider the mental health approach in university spaces.

It is necessary to emphasize that, although suicidal ideation is a risk factor associated with suicide, non-communication of suicidal thoughts is possible, suggesting that the prevention approaches need to be expanded to better predict young individuals at potential risk. Although it is not possible to establish that these young individuals are at greater risk of suicidal behavior in relation to other young people belonging to the same age group, who are not part of the university context, the data show high prevalence of this behavior in the population under study, presenting itself in a multifaceted way.

Since a considerable part of the student’s time is devoted to educational activities, it is urgent to turn the university environment into a health promoter capable of dealing with the identification of the mental health demands relevant to the moments experienced during the academic period. In view of such expressive data on suicidal behavior in young university students, further research studies are needed with larger samples and with application of more than one instrument to measure suicidal behavior, both for comparison purposes and to better understand the phenomenon in this population.
